# Effects of bacteriophage on *Salmonella* Enteritidis infection in broilers

**DOI:** 10.1038/s41598-023-38791-6

**Published:** 2023-07-27

**Authors:** Zahra Sarrami, Mohammad Sedghi, Ishmael Mohammadi, Mike Bedford, Hadi Miranzadeh, Razie Ghasemi

**Affiliations:** 1 Department of Animal Sciences, College of Agriculture, Isfahan University of Technology, Isfahan, 84156-83111 Iran; 2 AB Vista, Marlborough, SN8 4AN Wiltshire UK

**Keywords:** Animal physiology, Bacteriophages

## Abstract

Bacteriophages (BP) are viruses that can infect bacteria. The present study evaluated the effect of BP on *Salmonella* infected broilers. A number of 150 day-old broilers were used in a completely randomized design with five treatments that included: (1) basal diet from day 0 to 28; (2) basal diet + 0.3 g/kg of colistin from day 0 to 28; (3) basal diet from day 1 to 13, and basal diet + 0.4 g/kg of colistin from day 14 to 28; (4) basal diet + 1 g/kg of BP from day 0 to 28; (5) basal diet + 1.5 g/kg of BP from day 0 to 28. On day 13, 15 chickens from each treatment were challenged by *Salmonella* Enteritidis (SE), while fifteen from each treatment were not; instead, they were kept in the same cage with the challenged chickens (exposed chickens). At 7 and 14 days post-challenge, the number of SE and coliform bacteria in the cecum and liver of colistin and BP-fed birds was lower than the control treatment. In exposed and challenged chickens, the height and surface area of villus were greater in the BP and colistin-supplemented groups. Serum concentrations of aspartate aminotransferase and alanine transaminase were greater, while serum albumin and triglycerides concentrations were lower in the control treatment. The liver of the challenged chickens had more pathological lesions than exposed birds. BP significantly decreased *PPARγ* gene expression in exposed chickens. In the challenged and exposed chickens, *TLR4* gene expression was lower in BP and colistin-treated birds as compared to the control. In conclusion, adding BP to the diet from the day of age prevents the spread of *Salmonella*.

## Introduction

Salmonellosis is a zoonosis that can be easily transmitted from animals to humans through contaminated livestock products^1^. Salmonellosis, caused by *Salmonella* *enterica*, was first discovered by Salmon in 1885^2^. *Salmonella* is gram-negative, facultatively anaerobic bacteria that do not produce spores. They belong to the *Enterobacteriaceae* family, which inhabit the gastrointestinal tract (GIT) of birds and humans^3^; they can be considered as part of the normal GIT microflora in mammals and poultry^4^. The fecal material of birds is the major source of water and food contamination, which in turn is the main route for spreading *Salmonella* to the environment and, subsequently, the food supply chain^1^. A review of the data obtained from the Centers for Disease Control and Prevention (CDC), from 2006 to 2011, showed that 40% of *Salmonella* contamination was transmitted via poultry products such as meat and eggs^3^. Therefore, poultry and poultry products are considered the most important vehicles for *Salmonella* infections. Thus, controlling *Salmonella* in poultry is of great importance from a human health viewpoint^5^.

Antibiotics have been used since the 1940s to treat patients with a variety of bacterial diseases^6^. In veterinary medicine, the colistin antibiotic is used to prevent and treat some bacterial diseases, specifically those caused by gram-negative bacteria. The Committee for Medicinal Products for Veterinary Use (CVMP) has recommended colistin to treat *Salmonella*-induced GIT infections^7^ (it should be noted that this recommendation is only for the treatment of the disease and the temporary use of antibiotics). However, due to increased bacterial resistance, the use of colistin should be re-evaluated^8^. According to epidemiological data, Kempf et al. (2013) have reported that the use of colistin could lead to bacterial resistance in animals, which is transmitted to humans^9^. As a result, there is a great incentive to find alternatives to antibiotic use in the poultry industry^10^. As such, chemical additives such as organic acids and essential oils, or biological treatments such as probiotics and bacteriophages have been widely used to control *Salmonella* infection in poultry^11,12^.

Bacteriophages (BP) were discovered in 1900^13^. Bacteriophages are parasites of bacteria that multiply inside the bacterium using the host biosynthetic organelles^14^; they have been used to prevent and treat bacterial diseases. Bacteriophages have the ability to kill pathogenic bacteria. As such, they can be used as a safe alternative to antibiotics because they have no detrimental effects on eukaryotic cells^11^ and do not induce antibiotic resistance. Lytic BPs can be administered via drinking water and feed^15,16^. The use of BP to reduce pathogenic bacteria such as *Salmonella* in broilers and layers has been investigated in previous studies, most of which have confirmed that BP can control bacterial infections in poultry^17–21^.

Previous studies have also demonstrated that BP could remarkably inhibit inflammation at the humoral and cellular levels. The critical factors involved in this mechanism include pattern recognition receptors (PRRs) and pro-and anti-inflammatory genes^22–24^. It has also been shown that BPs up-regulate genes triggering cellular metabolism, reducing GIT permeability^25,26^. We hypothesized that dietary supplementation with BP could improve the growth performance of broilers by decreasing the inflammation in monocytes and improving metabolism in intestinal epithelial cells. Therefore, the objective of the present study was to evaluate the effects of BP on *Salmonella* infection, the microbial population of ceca, intestinal morphology, histopathological changes of the liver, some blood biochemical parameters, and the expression of peroxisome proliferator-activated receptor γ (*PPARγ*), Toll-like receptor 4 (*TLR4*)*,* and Interleukin 10 (*IL-10*) genes in the *Salmonella* challenged broiler chickens.

## Materials and methods

This experiment was conducted according to the comprehensive animal welfare guide, as adopted by FASS (2010) and all animal care and experimental procedures were approved by the Animal Policy and Welfare Committee of the Isfahan University of Technology. The authors confirm that they have adhered to the animal welfare statement’ in this manuscript and that all of the EU standards for the protection of animals and/or feed legislation have been met. Also, they confirm that all of the ARRIVE guidelines have been met.

## Experimental design

Room temperature was uniformly maintained at 33 ℃ for the first two days and then gradually reduced to 24 ℃ until 28 days. The lighting regime was 23L: 1D for the first three days and then reduced to 20L: 4D until the end of the experiment. All birds were fed the treatment diets ad libitum from 0 to 28 days of age (Table 1). A total of 150 day-old *as-hatched* broiler chickens (Ross 308) were used in a completely randomized design with five treatments and five replicates of six birds each. Dietary treatments included: (1) corn-soy based diet from day 0 to 28; (2) corn-soy based diet + 0.3 g/kg colistin antibiotic (from day 0 to 28); (3) corn-soy based diet from the beginning to d 13, followed by the corn-soy based diet with 0.4 g/kg of colistin added from d 14 (1 day post SE infection) to the end of the experiment (28 days); (4) corn-soy based diet + 1 g/kg of BP from 0 to 28 days; and (5) control diet corn-soy based diet + 1.5 g/kg of BP during 0–28 days. The BP used in this experiment was ProBe-Bac^®^ (Pathway Intermediates Company, Seoul, South Korea). ProBe-Bac is a BP cocktail (a mixture of several BPs), targeting *Salmonella* and *Escherichia* *coli* (*E.* *coli*) bacteria.

**Table 1:** Ingredient composition of the basal diets.

Diet composition (g/kg)			Analyzed composition		
Ingredients	Starter	Grower	Nutrients	Starter	Grower
Corn	518.2	563.7	Metabolizable energy (kcal/kg)	2985.0	3040.0
Soybean meal (CP 42%)	370.0	349.0	Crude protein (%)	23.00	20.90
Soybean oil	20.0	25.5	Digestible lysine (%)	1.28	1.15
Corn gluten meal (CP 60%)	50.0	25.0	Digestible methionine (%)	0.64	0.58
Salt	2.1	2.2	Digestible Met + Cys (%)	0.95	0.87
NaHCO_3_	2.3	2.2	Digestible threonine (%)	0.86	0.77
Di-calcium phosphate	16.5	13.8	Calcium (%)	0.96	0.87
Limestone	10.4	9.7	Available phosphorus (%)	0.48	0.43
Vitamin premix^a^	1.0	1.0			
Mineral premix^b^	1.0	1.0			
l-Lysine-HCL	3.4	2.5			
dl-Methionine	3.0	2.8			
l-Threonine	1.0	0.7			
Choline chloride	1.0	0.8			
Phytase 5000 (FTU/g)	0.1	0.1			

^a^ Supplied per kg of diet: 12,000 IU Vit A, 5000 IU Vit D3, 80 IU Vit E, 3.2 mg Vit K, 3.2 mg Vit B1, 8.6 mg Vit B2, 65 mg niacin, 20 mg pantothenic acid, 4.3 mg Vit B6, 0.22 mg biotin, 2.2 mg folic acid, 0.017 mg VitB12.

^b^ Supplied per kg of diet: 16 mg copper, 1.25 mg iodine, 20 mg iron, 120 mg manganese, 0.3 mg selenium, 110 mg zinc.

Also, on the 10th day of the experiment, the excreta from three birds in each cage (10% of all experimental birds) were sampled to confirm that all birds were *Salmonella* free. Briefly, feces of the birds were collected and used to inoculate a pure plate of XLD agar. After 24 h, no *Salmonella* (black colony) was found in the culture media. Thus, it was confirmed that the chickens were free of *Salmonella* at the beginning of the experiment and before they were challenged.

Chickens were challenged with *Salmonella* Enteritidis (SE) on day 13 to evaluate the effect of dietary treatments on infected birds. Bacteriologically and serologically avian strain of *Salmonella* *enterica* serotype Enteritidis were obtained from a reference laboratory for veterinary medicine from Tehran University. Concentrations of SE were verified by serial dilution and plated on brilliant green agar (BGA) to enumerate the actual colony-forming unit (CFU)^27^. On d 13, three birds from each cage were randomly selected and orally challenged with 0.5 mL of the SE culture suspended in the phosphate-buffered saline (PBS) at a 10^6^ CFU/mL concentration. The three remaining chickens from each cage were not challenged, but they were kept in the same cage with the challenged chickens and defined as the exposed chickens). The floor of each cage was covered with paper to expose all chickens to their excreta. 7 and 14 days after the challenge, 14 chickens from each treatment (seven challenged chickens and seven exposed chickens) were randomly selected and humanely killed for sampling.

### Microbial culture

Fourteen chickens from each treatment (seven challenged chickens and seven exposed chickens) were euthanized at 7 and 14 days post-challenge (DPC) for isolation of SE and counting of coliform bacteria from the cecum contents and liver. One g of the cecum content and/or liver tissue was macerated in 9 mL of the peptone water broth^28^. One hundred µL of the solution was then homogenized in 900 µL PBS and serially (1:10) diluted. The final dilutions were prepared in PBS and 100 µL of each dilution was inoculated onto a BGA plate (10 g/L protease peptone, 3 g/L yeast extract, 10 g/L lactose, 10 g/L sucrose, 5 g/L sodium chloride, 0.08 g/L phenol red, 12.5 mg/L brilliant green, 12 g/L agar; pH  6.9 ± 0.2). These plates were incubated for 48 h at 37 ℃ and the pink colonies were counted as *Salmonella*^29,30^. To count coliform bacteria, 100 µL of each dilution was inoculated onto MacConkey agar plates (17 g/L peptone from casein, 3 g/L peptone from meat, 5 g/L sodium chloride, 10 g/L lactose, 1.5 g/L bile salt mixture, 0.03 g/L neutral red, 0.001 g/L crystal violet, and 13.5 g/L agar; pH  7.1 ± 0.2). These plates were incubated for 24 h at 37 ℃^31^. The results were reported as log 10 of CFU per gram of digesta.

### Measurements of blood biochemical parameters

Fourteen blood samples from each treatment (seven challenged chickens and seven exposed chickens) were obtained from the wing vein of the birds at seven DPC. The serum was isolated and stored at −20 ℃ pending further analyses. Serum concentrations of total protein, albumin, cholesterol, triglyceride (TG), HDL, LDL, aspartate aminotransferase (AST), and alanine transaminase (ALT) were measured by the commercial kits (Pars Azmun, Iran) and autoanalyzer (Alcyon, American). Serum globulins were calculated by subtracting the serum albumin levels from the total serum protein values^32^.

### Intestinal morphological analysis

For enteric morphology analysis, at 7 and 14 DPC, 14 jejunal samples from each treatment (seven challenged chickens and seven exposed chickens) were collected. One cm of the jejunaʼs midpoint from each bird was removed and fixed in 10% buffered formalin^33^. Samples were prepared as described by Ekim et al.^34^ for evaluation by an optical microscope (Olympus CX31, Tokyo, Japan) and photographed with a digital microscope camera. Crypt depth, height, and width of the villi, and muscular layers thickness were measured using the ImageJ software^34,35^. The formula used to calculate the villus surface area was 2π × (villus width/2) × villus height^36^.

### Liver histopathology

At 14 DPC, one cm liver section was removed from fourteen chickens (seven challenged and seven exposed chickens) and prepared for evaluation under a microscope, as described by Babinska et al., 2012 and Garcia et al., 2010^37,38^. Briefly, one cm of liver tissue was fixed in 10% formalin and embedded in paraffin blocks, then sections of 5 µm thickness were stained with hematoxylin–eosin. Slides were examined using an optical microscope (Carl Zeiss, Jena), and a variety of liver lesions were observed. Liver lesion scores were determined as follows: score + 1: mild pathological change, score + 2: moderate pathological change, and score + 3: hyper-pathological change.

### Total RNA extraction and real-time quantitative PCR

At 14 DPC, two cm of the midpoint of the ileum from each slaughtered chicken was obtained for the gene expression analysis. All samples were instantly frozen in liquid nitrogen. Primers were designed based on the target gene sequences and blasted with the NCBI Blast Primer. the primers were synthesized commercially (TAG Copenhagen, Denmark, Table 2). RNA extraction was performed using TRIzol Reagent (Sinaclon) according to the manufacturer’s guidelines. Complementary DNA (cDNA) was synthesized from the total RNA using cDNA synthesis^®^ RT reagent Kit (Sinaclon). For each sample, 25 ng of cDNA was used as a template in a 25 μL final reaction according to the manufacturer’s protocol. The expression of three candidate genes, including *PPARγ*, *TLR4* and *IL-10* was determined using quantitative reverse transcription-PCR (qRT-PCR, ABI StepOne™ Real-Time PCR System—Thermo Fisher Scientific. The reaction was performed using RealQ Plus 2× Master Mix Green (Amplicon). The thermal cycling conditions consisted of an initial denaturation step at 95 ℃ for 10 min; this was followed by 40 cycles, including the denaturation step at 95 ℃ for 30 s, and an annealing and extension step at 60 ℃ for 30 s. The *GAPDH* gene was used as an internal control. Each experiment, performed in triplicate, was repeated three times independently. The cycle threshold (Ct) values of the triplicate PCRs were averaged and the relative quantification of the transcript levels was performed using the comparative 2^−ΔΔCT^ method. The fold change in the target gene, relative to *GAPDH,* was determined according to the following formula: fold change = 2^−ΔΔCT^, where ΔΔCT = (Ct target gene − Ct *GAPDH*).

**Table 2:** Specific amplification of the gene and internal reference primer.

Gene	Forward primer (5′–3′)	Reverse primer (5′–3′)	References
*GAPDH*	GAAGCTTACTGGAATGGCTTTCC	CGGCAGGTCAGGTCAACAA	^100^
*PPARγ*	CACTGCAGGAACAGAACAAAGAA	TCCACAGAGCGAAACTGACATC	^101^
*TLR4*	AGTCTGAAATTGCTGAGCTCAAAT	GCGACGTTAAGCCATGGAAG	^102^
*IL-10*	CTGTCACCGCTTCTTCACCT	ACTCCCCCATGGCTTTGTA	^103^

### Statistical analyses

Data were analyzed using the GLM procedures of SAS 9.4 statistical software (SAS Institute, 1999) as a completely randomized design (CRD). An ANOVA was performed where treatment was used as the independent and the parameter of interest as the dependent. Treatment means were separated using Tukey’s tests with significance differences declared at *P* < 0.05. Additionally, the linear and quadratic effects of feeding control, 1 g BP and 1.5 g BP treatments were analyzed using polynomial contrasts. Furthermore, an orthogonal contrast was employed to test for differences between the control and the average of the two BP-supplemented birds.

### Ethics approval

Animal welfare statement: The authors confirm that they have adhered to the animal welfare statement’ in this manuscript, and they confirm that all of the EU standards for the protection of animals and/or feed legislation have been met. The only exception was for stock density; in this case, the final body weight was set to be less than 30 kg/m^2^, which was lower than that mentioned in the council directive 2007/43/EC of June 28, 2007. We also confirm that we have followed the animal welfare guide, as adopted by FASS (2010). All animal care and experimental procedures were approved by the animal policy and welfare committee of Isfahan University of Technology. Also, this study followed the ARRIVE guidelines.

## Results

### Microbial culture

**Figure 1:**
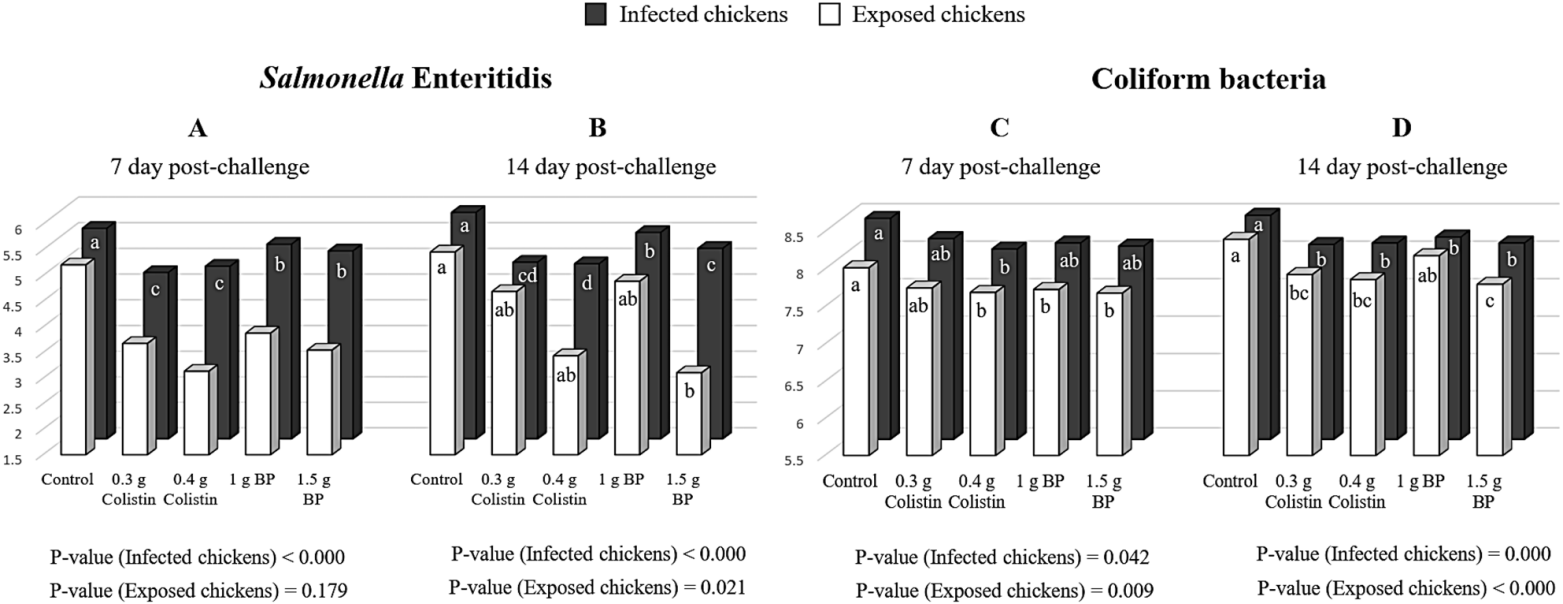
Re-isolation of *Salmonella* Enteritidis and counting the coliform bacteria from the cecal contents at 7 and 14 day post-challenge (Log10 CFU/g). 0.3 g colistin: 0.3 g/kg colistin in diet; 0.4 g colistin: 0.4 g/kg colistin in diet; 1 g BP: 1 g/kg bacteriophage in diet; 1.5 g BP: 1.5 g/kg bacteriophage in diet. Superscript abcd: Values followed by different letters in each factor are significantly different. *P* < 0.05; Tukey's pairwise test.

The results of SE and coliform bacteria enumeration from the caecal contents are shown in Fig. 1 A to D and Table 3. At seven DPC (Fig. 1A), in the infected chickens, colistin-fed birds had less SE in comparison to the other treatments (*P* < 0.05). At 14 DPC (Fig. 1B), the lowest number of SE in the infected chickens was related to colistin treatments and 1.5 g BP; meanwhile, in the exposed chickens, the lowest number of SE was observed in the birds fed the diet containing 1.5 g/kg BP. At seven DPC (Fig. 1C), the control and 0.4 g colistin-fed challenged birds had the highest and lowest number of coliform bacteria, respectively (*P* < 0.05). The lowest number of coliform bacteria in the exposed chickens was observed in the BP-fed and 0.4 g colistin-fed birds (*P* < 0.05). At 14 DPC (Fig. 1D), in the infected and exposed chickens, the number of coliform bacteria in the control group was significantly higher than that in the other treatments, except for 1 g BP-fed birds (*P* < 0.05). At seven and 14 DPC (Table 3), in both infected and exposed birds, the number of SE and coliform bacteria in the cecum of BP-treated chickens was lower than that in the control birds (*P* < 0.05). Also, no difference was observed in the number of the cecal coliform bacteria and SE in the exposed chickens between BP and colistin-treated chickens at seven and 14 DPC (*P* < 0.05). In the infected and exposed birds, increasing the dietary BP level reduced the number of SE and coliform bacteria in the cecum linearly (*P* < 0.05).

**Table 3:** Orthogonal and polynomial contrast analysis for counting of SE and coliform bacteria from cecum and liver at 7 and 14 day post-challenge.

P-value^1^	*Salmonella* Enteritidis				Coliform bacteria			
	7 day post-challenge		14 day post-challenge		7 day post-challenge		14 day post-challenge	
	Infected chickens	Exposed chickens	Infected chickens	Exposed chickens	Infected chickens	Exposed chickens	Infected chickens	Exposed chickens
Cecum								
Control vs BP	< 0.000	0.027	< 0.000	0.021	0.004	0.002	< 0.000	0.002
BP vs Colistin	< 0.000	0.653	< 0.000	0.911	0.898	0.830	0.545	0.318
Lin	< 0.000	0.026	< 0.000	0.004	0.005	0.002	< 0.000	0.000
Quad	0.744	0.722	0.358	0.104	0.437	0.477	0.099	0.160
Liver								
Control vs BP	0.063	0.012	0.034	0.006	< 0.000	0.027	0.000	< 0.000
BP vs Colistin	0.697	0.486	0.973	0.918	0.110	0.827	0.000	0.450
Lin	0.022	0.004	0.015	0.005	< 0.000	0.023	< 0.000	< 0.000
Quad	0.251	0.385	0.474	0.861	0.015	0.818	0.003	0.057

^1^Control vs BP: contrasting birds not treated with BP or colistin versus birds treated with BP; BP vs colistin: contrasting birds treated with BP versus birds treated with colistin; Lin: linear effects of increasing inclusion levels of BP; Quad: quadratic effects of increasing inclusion levels of BP.

**Figure 2:**
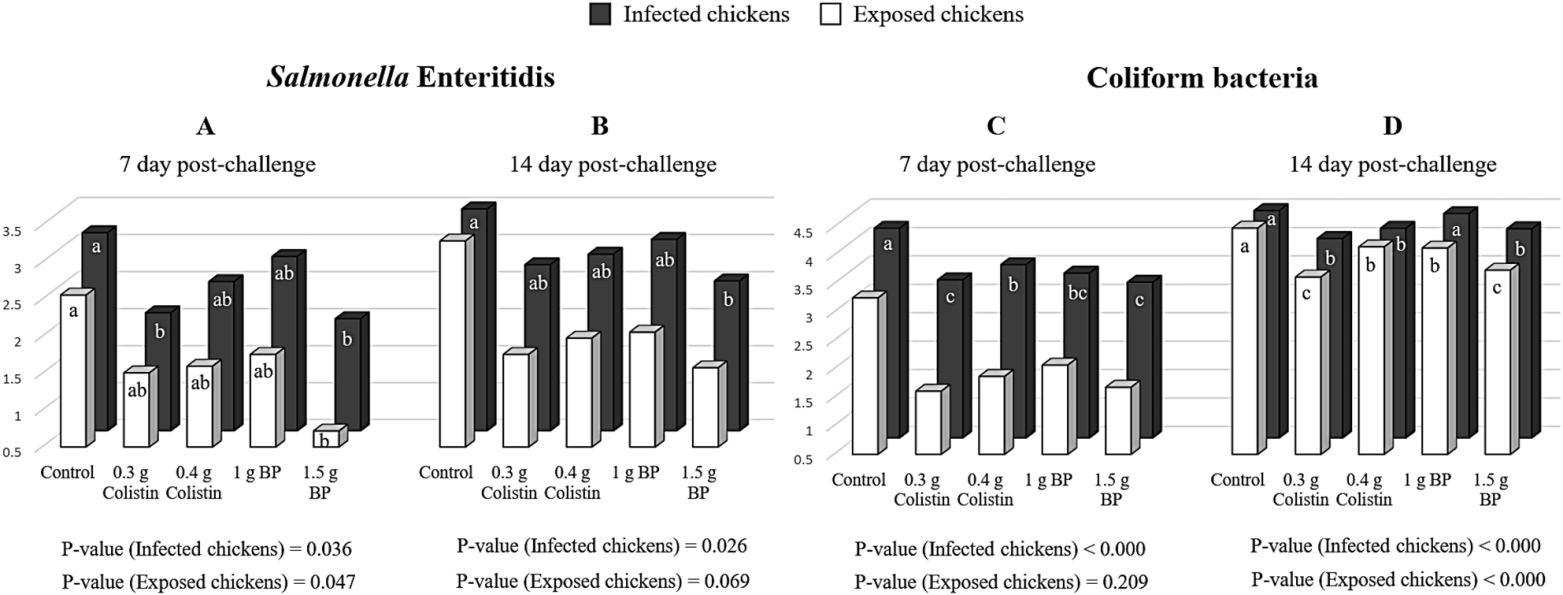
Re-isolation of *Salmonella* Enteritidis and counting of coliform bacteria from the **liver** at 7 and 14 day post-challenge (Log10 CFU/g). 0.3 g colistin: 0.3 g/kg colistin in diet; 0.4 g colistin: 0.4 g/kg colistin in diet; 1 g BP: 1 g/kg bacteriophage in diet; 1.5 g BP: 1.5 g/kg bacteriophage in diet. Superscript abc: values followed by different letters, are significantly different. *P* < 0.05; Tukey's pairwise test.

The results of isolating SE and counting coliform bacteria from the liver are shown in Fig. 2A–D and Table 3. At 7 DPC (Fig. 2A), in the challenged and exposed chickens, the number of SE in 1.5 g BP birds was lower than that in the control birds (*P* < 0.05). At 14 DPC (Fig. 2B), the lowest SE number in the infected chickens was found in the birds fed the diet containing 1.5 g/kg BP. In the exposed chickens, the SE number was similar between treatments. At 7 DPC (Fig. 2C), in the infected chickens, the number of coliform bacteria in the control group was significantly higher than that in the other treatments, with the least number of coliform bacteria being found in the 1.5 g/kg BP and 3 g/kg colistin-fed birds. In the exposed chickens, no difference was found among the treatments with regard to the number of coliform bacteria. At 14 DPC (Fig. 2D), in the challenged chickens, the number of coliform bacteria in the chickens fed with both colistin groups and 1.5 g BP was significantly lower than that in the other treatments (*P* < 0.05). Also, in the exposed chickens, the number of coliform bacteria in 3 g/kg colistin and 1.5 g/kg BP treatments was lower than that in the other treatments (*P* < 0.05). At 7 and 14 DPC (Table 3), the supplemental BP in the exposed and challenged chickens reduced the number of SE and coliform bacteria in the liver, as compared to the control treatment (*P* < 0.07). However, no significant difference was observed in the chickens supplemented with BP and colistin in terms of SE and coliform bacteria counts of the liver (P < 0.05). Also, in the infected and exposed birds, with increasing the dietary BP level, the number of SE and coliform bacteria in the liver was reduced linearly (*P* < 0.05).

### Intestinal morphological changes

**Figure 3:**
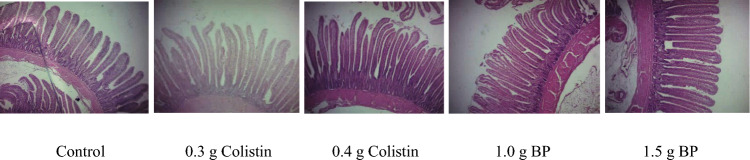
Effect of BP and colistin on jejunum morphology in infected chickens at 14 day post-challenge. 0.3 g colistin: 0.3 g/kg colistin in diet; 0.4 g colistin: 0.4 g/kg colistin in diet; 1 g BP: 1 g/kg bacteriophage in diet; 1.5 g BP: 1.5 g/kg bacteriophage in diet.

The effects of adding BP and colistin to the diets on the jejunum histological changes in the infected chickens are shown in Table 4 and Fig. 3. At 7 DPC, the highest and lowest villus height, villus height to crypt depth ratio, and villus surface area were related to 1.5 g BP and control treatments, respectively (*P* < 0.05). There were no significant differences among the treatments in terms of crypt depth, villus width, and muscle thickness. At 14 DPC, the highest and lowest crypt depth was observed in the control and 1.5 g/kg BP-supplemented birds, respectively (*P* < 0.05). Supplementing birds with BP and colistin increased the villus height to crypt depth ratio (*P* < 0.05). Villus surface area, villus width and muscle thickness were similar among treatments. The addition of BP increased villus height, villus height to crypt depth ratio and villus surface area linearly (*P* < 0.05).

**Table 4:** Effect of adding BP and colistin to the diets on histological changes of jejunum in infected chickens.

Morphometric parameters	Dietary treatments					SEM	P-value^1^			
	Control	0.3 g colistin	0.4 g colistin	1.0 g BP	1.5 g BP		Trt	C vs BP	Lin	Quad
7 day post-challenge										
Villus height (µm)	682.56^b^	744.20^ab^	733.24^ab^	798.55^ab^	827.15^a^	34.609	0.049	0.009	0.008	0.671
Crypt depth (µm)	92.85	88.73	90.94	89.76	79.74	7.151	0.732	0.381	0.260	0.546
VH: CD	7.58^b^	8.66^ab^	8.15^ab^	9.24^ab^	10.54^a^	0.585	0.013	0.009	0.004	0.703
Villus width (µm)	90.12	95.37	90.71	95.69	97.52	3.118	0.372	0.064	0.058	0.852
VSA (mm^2^)	0.19^b^	0.22^ab^	0.20^ab^	0.23^ab^	0.25^a^	0.012	0.017	0.002	0.002	0.648
Muscle thickness (µm)	203.24	231.67	221.90	230.83	231.22	13.680	0.539	0.072	0.086	0.554
14 day post-challenge										
Villus height (µm)	823.58^b^	999.82^a^	851.27^b^	846.04^b^	919.74^ab^	34.864	0.007	0.161	0.074	0.327
Crypt depth (µm)	115.01^a^	109.65^ab^	104.61^ab^	95.52^bc^	92.09^c^	4.078	0.001	0.000	0.000	0.437
VH: CD	7.23^c^	9.19^ab^	8.16^bc^	8.87^abc^	10.07^a^	0.424	0.000	0.000	0.000	0.639
Villus width (µm)	110.14	116.31	111.98	111.98	113.12	5.838	0.960	0.743	0.724	0.984
VSA (mm^2^)	0.28	0.36	0.29	0.29	0.32	0.020	0.066	0.333	0.225	0.564
Muscle thickness (µm)	215.41	257.67	233.02	218.55	229.58	13.785	0.236	0.489	0.369	0.619

0.3 g colistin: 0.3 g/kg colistin in diet; 0.4 g colistin: 0.4 g/kg colistin in diet; 1 g BP: 1 g/kg bacteriophage in diet; 1.5 g BP: 1.5 g/kg bacteriophage in diet.

*VH:* *CD* villus height to crypt depth ratio; *VSA* villus surface area.

^1^Trt: overall effects of treatments; C vs BP: contrasting birds not treated with BP or colistin versus birds treated with BP; Lin: linear effects of increasing inclusion levels of BP; Quad: quadratic effects of increasing inclusion levels of BP.

^abc^Values within a row followed by different superscripts are significantly different. *P* < 0.05; Tukey's pairwise test.

**Figure 4:**
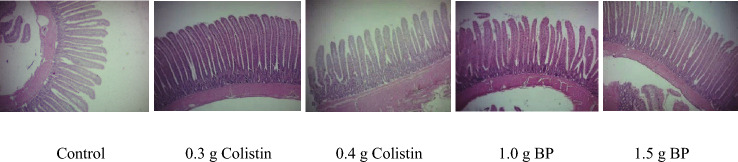
Effect of BP and colistin on jejunum morphology in exposed chickens at 14 day post-challenge. 0.3 g colistin: 0.3 g/kg colistin in diet; 0.4 g colistin: 0.4 g/kg colistin in diet; 1 g BP: 1 g/kg bacteriophage in diet; 1.5 g BP: 1.5 g/kg bacteriophage in diet.

The effects of supplementing birds with BP and colistin on the histological changes of the jejunum in the exposed chickens are shown in Table 5 and Fig. 4. At 7 DPC, the lowest villus height was related to the control and 0.3 g colistin treatments. Villus height to crypt depth ratio was increased in the chickens supplemented with colistin and BP, compared to the control birds (*P* < 0.05). Also, the villus surface area in the birds supplemented with 4.0 g/kg colistin and 1.5 g/kg BP was greater than the other birds (*P* < 0.05). At 14 DPC, dietary supplementation with 3.0 g/kg colistin and 1.5 g/kg BP resulted in a greater muscle thickness (*P* < 0.05). Dietary treatments had no effect on the villus height, crypt depth, villus height to crypt depth ratio, villus width and villus surface area. Supplementing chickens with BP reduced the crypt depth, while it increased the villus height to crypt depth ratio linearly (*P* < 0.05).

**Table 5:** Effect of adding BP and colistin to the diets on histological changes of jejunum in exposed chickens.

Morphometric parameters	Dietary treatments					SEM	P-value^1^			
	Control	0.3 g colistin	0.4 g colistin	1.0 g BP	1.5 g BP		Trt	C vs BP	Lin	Quad
7 day post-challenge										
Villus height (µm)	621.00^b^	636.90^b^	838.30^a^	761.20^ab^	799.30^ab^	44.359	0.003	0.023	0.022	0.748
Crypt depth (µm)	92.52^a^	71.39^b^	90.29^a^	89.95^a^	82.56^ab^	4.233	0.008	0.258	0.152	0.463
VH: CD	6.65^b^	8.90^a^	9.46^a^	8.51^ab^	9.78^a^	0.511	0.001	0.001	0.000	0.750
Villus width (µm)	90.54	85.41	91.88	91.54	97.28	4.053	0.379	0.328	0.191	0.406
VSA (mm^2^)	0.17^b^	0.17^b^	0.24^a^	0.21^ab^	0.24^a^	0.014	0.001	0.009	0.006	0.978
Muscle thickness (µm)	200.80^ab^	171.70^b^	249.90^a^	250.40^a^	255.00^a^	15.881	0.001	0.025	0.029	0.545
14 day post-challenge										
Villus height (µm)	794.43	917.87	812.42	841.58	945.81	43.302	0.077	0.105	0.048	0.376
Crypt depth (µm)	108.47	99.63	100.20	101.84	100.19	4.964	0.702	0.244	0.241	0.862
VH: CD	7.39	9.32	8.19	8.40	9.57	0.581	0.079	0.049	0.025	0.568
Villus width (µm)	108.30	112.48	108.82	108.74	111.95	4.676	0.946	0.699	0.592	0.712
VSA (mm^2^)	0.26	0.32	0.27	0.28	0.33	0.019	0.104	0.112	0.050	0.355
Muscle thickness (µm)	185.68^b^	254.90^a^	195.82^b^	236.45^ab^	251.70^a^	12.389	0.000	0.001	0.000	0.665

0.3 g colistin: 0.3 g/kg colistin in diet; 0.4 g colistin: 0.4 g/kg colistin in diet; 1 g BP: 1 g/kg bacteriophage in diet; 1.5 g BP: 1.5 g/kg bacteriophage in diet.

*VH:* *CD* villus height to crypt depth ratio; *VSA* villus surface area.

^1^Trt: overall effects of treatments; C vs BP: contrasting birds not treated with BP or colistin versus birds treated with BP; Lin: linear effects of increasing inclusion levels of BP; Quad: quadratic effects of increasing inclusion levels of BP.

^abc^Values within a row followed by different superscripts are significantly different. *P* < 0.05; Tukey's pairwise test.

### Liver pathological changes

**Figure 5:**
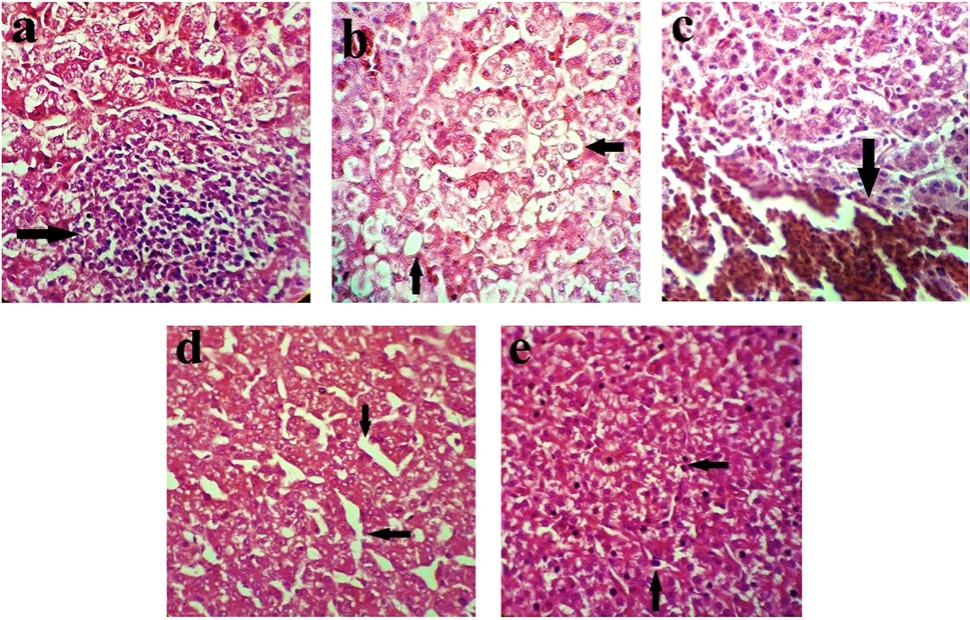
Photomicrography of liver histological: superscript a: hepatitis with Immune cell infiltration (arrow) and cloudy swelling of hepatocytes; superscript b: fat degeneration and vacuolar changes; superscript c: hemorrhages; superscript d: normal structure change and Remac separation; superscript e: necrosis.

The effects of adding BP and colistin on the pathological changes of the liver at 14 DPC are shown in Table 6 and Fig. 5. In the infected chickens**,** mild immune cell infiltration and necrosis with the cloudy swelling of hepatocytes could be evidently seen in the control group. At the same time, fewer birds in the colistin and BP treatments had hepatitis, necrosis and cloudy swelling of hepatocytes. In the control treatment, the necrotic areas in the liver were more extensive; they were infiltrated with heterophils, lymphocytes and macrophages. Congestion with a score of + 1 was observed in all birds’ livers (congestion is a localized increase of blood in a specific tissue). However, no bleeding was observed in the liver of the birds treated with colistin and BP, but 14% of the birds in the control treatment had bleeding in their liver. The liver of the birds fed with BP had a normal structure; despite this, the liver structure of the control birds had been changed and the Remac plates were spaced apart. All infected chickens had fat degeneration lesions. However, the histopathological lesion score in the control birds was + 3, while in the other treatments, the liver had a fat degeneration lesion with a score + 2 and + 1.

**Table 6:** Effect of BP and colistin on pathological change of liver at 14 day post-challenge.

Pathological parameters	Infected chickens					Exposed chickens				
	Control	0.3 g colistin	0.4 g colistin	1.0 g BP	1.5 g BP	Control	0.3 g colistin	0.4 g colistin	1.0 g BP	1.5 g BP
Percent of birds showing lesions of various scores (%)										
Necrosis	57	0	43	43	14	14	0	0	14	0
Hepatitis	71	57	29	57	57	43	43	14	43	14
Fat degeneration	100	100	100	100	100	100	57	100	86	71
Congestion^a^	100	100	100	100	100	100	100	100	100	100
Hemorrhages	14	0	0	0	0	14	0	14	0	0
Swelling of hepatocytes	100	100	100	100	100	71	29	14	57	57
Remac separation	29	0	14	0	0	0	0	0	0	0
Histopathological lesion scores^b^										
Necrosis	+ 1		+ 1	+ 1	+ 1	+ 1			+ 1	
Hepatitis	+ 1	+ 1	+ 1	+ 1	+ 1	+ 1	+ 1	+ 1	+ 1	+ 1
Fat degeneration	+ 3	+ 1	+ 2	+ 2	+ 2	+ 1	+ 1	+ 1	+ 2	+ 1
Congestion	+ 1	+ 1	+ 1	+ 1	+ 1	+ 1	+ 1	+ 1	+ 1	+ 1
Hemorrhages	+ 1					+ 1		+ 1		
Swelling of hepatocytes	+ 2	+ 1	+ 1	+ 1	+ 1	+ 1	+ 1	+ 1	+ 1	+ 1
Remac separation	+ 1		+ 1							

0.3 g colistin: 0.3 g/kg colistin in diet; 0.4 g colistin: 0.4 g/kg colistin in diet; 1 g BP: 1 g/kg bacteriophage in diet; 1.5 g BP: 1.5 g/kg bacteriophage in diet.

^a^Congestion is a localized increase of blood in a particular tissue.

^b^Score + 1: mild pathological change; score + 2: moderate pathological change; score + 3: hyper pathological change.

In the exposed chickens, mild immune cell infiltration and cloudy swelling of hepatocytes were evident in all birds; however, only a few birds in the colistin and BP treatments had swelling of hepatocytes lesion, and only 14% of the birds treated by the supplementation with 1.5 g/kg BP and 4.0 g/kg colistin had hepatitis. Necrotic lesions were not observed in the birds supplemented with colistin and 1.5 g/kg BP; however, 14% of the birds in the control treatment and 1.0 g/kg BP had necrotic lesions with a score of + 1. Congestion with a score of + 1 was observed in all livers. No bleeding was, however, observed in the liver of the birds supplemented with BP, but 14% of the birds in the control and 4.0 g/kg colistin groups had bleeding in their liver. All birds except those in the 1.0 g/kg BP treatment had fat degeneration lesion with a score of + 1, but a higher percentage of the control birds had this lesion. Normal structure change and Remac separation were not observed in any treatment.

### Blood biochemical parameters

The effects of adding BP and colistin on blood parameters at 7 DPC are shown in Table 7. Serum concentrations of aspartate aminotransferase (AST) and alanine transaminase (ALT) in the control treatment were greater than those in the other treatments; this difference was significant for AST in the infected chickens (*P* < 0.05) and for ALT in the exposed chickens (*P* < 0.05). Also, in the infected chickens, BP reduced the serum concentration of AST linearly (*P* < 0.05). There was no significant difference among the treatments with regard to the serum concentrations of the total protein and albumin in the infected chickens; however, in the exposed chickens, BP supplementation increased the serum concentration of albumin and albumin to globulin ratio linearly (*P* < 0.05). Also, there was no significant difference in the serum globulin level among challenged chickens; despite this, in the exposed chickens, the greatest and lowest globulin levels were observed in the control and 3.0 g/kg of the colistin treatments, respectively (*P* < 0.05). In the challenged and exposed chickens, the albumin to globulin ratio in the 3.0 g/kg colistin-fed birds was greater than that in the control treatment (*P* < 0.05). In the challenged chickens, the serum concentration of TG in the 3.0 g/kg colistin-fed birds was greater than that in the control treatment (*P* < 0.05). Also, BP increased TG linearly (*P* < 0.05). In the challenged birds, there were no significant differences in terms of cholesterol, HDL and LDL levels; also, in the exposed chickens, the serum concentrations of cholesterol, TG, HDL and LDL were similar among birds.

**Table 7:** Effect of BP and colistin on blood biochemical parameters at 7 day post-challenge.

Treatments	AST (U/L)	ALT (U/L)	T.Pr (g/dl)	Albumin (g/dl)	Globulin (g/dl)	A/G ratio	TG (mg/dl)	Cholesterol (mg/dl)	HDL (mg/dl)	LDL (mg/dl)
Infected chickens										
Control	200.57^a^	6.28	2.95	1.22	1.72	0.73^b^	49.00^b^	106.71	48.28	38.71
0.3 g colistin	125.71^b^	5.28	2.95	1.58	1.42	1.06^a^	61.00^a^	127.57	57.28	46.71
0.4 g colistin	133.29^b^	5.42	3.20	1.35	1.68	0.89^ab^	50.00^b^	123.28	49.14	54.14
1.0 g BP	155.14^b^	5.57	2.85	1.25	1.64	0.77^ab^	56.71^ab^	111.14	52.28	36.71
1.5 g BP	151.29^b^	5.57	3.04	1.30	1.62	0.87^ab^	57.71^ab^	112.14	53.57	36.00
SEM	7.607	0.543	0.117	0.095	0.092	0.070	2.678	5.502	2.532	6.420
P-value^1^										
Trt	< 0.000	0.732	0.335	0.089	0.213	0.026	0.013	0.057	0.113	0.234
C vs BP	0.000	0.361	0.966	0.689	0.393	0.253	0.028	0.300	0.242	0.722
Lin	0.000	0.387	0.784	0.635	0.398	0.163	0.031	0.299	0.231	0.714
Quad	0.219	0.762	0.362	0.880	0.862	0.564	0.594	0.865	0.904	0.977
Exposed chickens										
Control	152.14	6.42^a^	2.84	1.20	1.64^a^	0.76^b^	68.14	123.14	53.71	45.71
0.3 g colistin	143.42	4.00^b^	2.63	1.30	1.22^b^	1.10^a^	53.71	122.57	51.85	49.42
0.4 g colistin	148.00	5.28^ab^	3.02	1.40	1.63^a^	0.90^ab^	61.86	106.28	45.00	31.14
1.0 g BP	149.71	5.57^ab^	2.85	1.31	1.46^ab^	0.94^ab^	56.28	119.28	50.71	46.71
1.5 g BP	145.00	5.00^ab^	2.88	1.41	1.43^ab^	1.00^ab^	54.71	112.92	44.57	47.14
SEM	3.186	0.462	0.099	0.065	0.095	0.067	4.796	5.019	5.211	5.226
P-value^1^										
Trt	0.324	0.015	0.123	0.162	0.026	0.019	0.205	0.114	0.642	0.597
C vs BP	0.233	0.086	0.822	0.021	0.119	0.008	0.051	0.302	0.435	0.739
Lin	0.148	0.064	0.782	0.011	0.121	0.007	0.055	0.218	0.339	0.741
Quad	0.562	0.883	0.912	0.672	0.769	0.779	0.643	0.666	0.694	0.948

0.3 g colistin: 0.3 g/kg colistin in diet; 0.4 g colistin: 0.4 g/kg colistin in diet; 1 g BP: 1 g/kg bacteriophage in diet; 1.5 g BP: 1.5 g/kg bacteriophage in diet.

*AST* aspartate aminotransferase, *ALT* alanine transaminase, *T.Pr* total protein, *A/G* albumin to globulin ratio, *TG* triglyceride, *HDL* high-density lipoprotein, *LDL* low-density lipoprotein.

^ab^Values within a column followed by different superscripts are significantly different. *P* < 0.05; Tukey's pairwise test.

^1^Trt: overall effects of treatments; C vs BP: contrasting birds not treated with BP or colistin versus birds treated with BP; Lin: linear effects of increasing inclusion levels of BP; Quad: quadratic effects of increasing inclusion levels of BP.

### Intestinal-related gene expression

**Figure 6:**
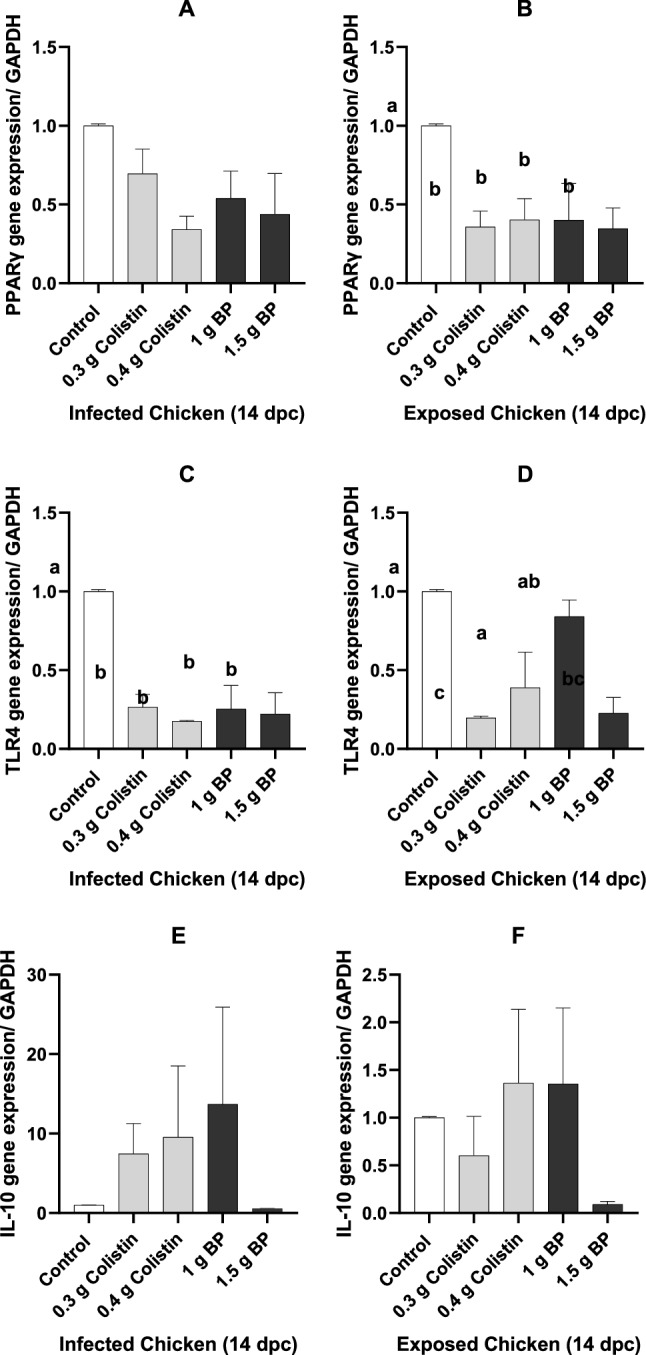
Gene expression in the exposed and infected chickens 14 day after infection. 0.3 g colistin: 0.3 g/kg colistin in diet; 0.4 g colistin: 0.4 g/kg colistin in diet; 1 g BP: 1 g/kg bacteriophage in diet; 1.5 g BP: 1.5 g/kg bacteriophage in diet. (**A**) *PPARγ* gene expression in the infected chicken. (**B**) *PPARγ* gene expression in the exposed chicken. (**C**) *TLR4* gene expression in the infected chicken. (**D**) *TLR4* gene expression in the exposed chicken. (**E**) *IL-10* gene expression in the infected chicken. (**F**) *IL-10* gene expression in the exposed chicken.

The relative mRNA expression of the intestinal genes in the infected and exposed chickens is presented in Fig. 6A–F. At 14 DPC, the SE challenge considerably changed the expression of peroxisome proliferator-activated receptor-γ (*PPARγ*) and toll-like receptor 4 (*TLR4*) (*P* < 0.05). In the infected chickens, *PPARγ* gene expression was not affected by colistin and BP groups, as compared to the control (*P* < 0.05; Fig. 6A). In the exposed chickens, feeding birds with diets containing BP and colistin significantly decreased the gene expression of *PPARγ,* as compared to the control (*P* < 0.05; Fig. 6B). In the infected chickens, *TLR4* gene expression was significantly lower in the colistin and BP groups than in the control group (*P* < 0.05; Fig. 6C). Meanwhile, in the exposed chickens, the gene expression of *TLR4* in the 1.5 g BP and 0.3 g/kg colistin groups was lower than that in the other groups (*P* < 0.05; Fig. 6D). In the infected chickens, the greatest and lowest level of *IL-10* gene expression was numerically achieved by dietary supplementation with BP at the level of 1 and 1.5 g/kg, respectively (*P* > 0.05), while colistin supplementation at the level of 0.3 and 0.4 g/kg led to the increased *IL-10* gene expression as compared to the control (*P* > 0.05; Fig. 6E). Furthermore, the lowest numerical expression of *IL-10* in the exposed chickens was related to birds fed by the 1.5 g/kg BP containing diet (*P* < 0.05; Fig. 6F). In addition, group contrast analysis between BP and control groups for the three candidate genes demonstrated that *PPARγ* transcription was significantly decreased in the exposed chickens (*P* = 0.0091). However, in the infected chicken, there was only a tendency for reduced *PPARγ* transcription (*P* = 0.0595). *TLR4* expression was significantly reduced in both infected (*P* = 0.0022) and exposed chickens (*P* = 0.0049) compared with the control. Lastly, the numerical increase in *IL-10* transcription in the infected (*P* = 0.4068) and exposed chickens (*P* = 0.5548) was not significant (Fig. 6E, F).

## Discussion

Bacteriophages are viruses that specifically infect bacteria^39,40^. The specific function of BP is important to us because BP therapy causes less damage to the normal microflora of the GIT as compared to the growth-promoting antibiotics, which can often cause damage to the normal microbiota, leading to secondary infections^41^. In this study, we used a BP cocktail (a mixture of several BPs) targeting *Salmonella* and *E.* *coli* bacteria. We found that BP was effective in treating SE and reducing its population in the cecum where it is known to colonize first. It has been shown that lytic BP can be used in poultry diets to prevent or treat *Salmonella* infection^18,20,42,43^. Fiorentin et al. (2005) used a mixture of BPs for treating SE, reporting that the use of such BPs is a suitable strategy for treating bacterial disease^44^. Further supporting evidence of the value of phage therapy for reducing *Salmonella* infection in poultry is provided by other authors^17,19,30^.

Also, our results showed that the population of coliform bacteria in the cecum of the birds supplemented with BP and colistin was lower than that in the control group. One reason for the reduction of coliform bacteria is the reduction of *Salmonella* in the cecum of birds by phage and colistin, which has increased the health and maintained the stable conditions of the digestive tract of birds that were treated with phage and colistin^45^. Also, because this phage cocktail has an effect on *E.* *coli*, another reason for the decrease in coliform bacteria is probably the decrease in *E.* *coli*.

Villus height and villus height to crypt depth ratio were also increased in birds fed with BP or colistin supplemented diets, which is likely due to reduced numbers of pathogenic bacteria in the intestine^46^. Previous work has indeed suggested that controlling *Salmonella* populations coincided with improved intestinal morphological structure and bird performance^31^ which is beneficial for performance. Intestinal morphology is a good indicator of the GIT health status and its response to the use of certain foods. The rapid maturation and development of the GIT can provide a good place for bacteria to colonize, and an increase of beneficial bacteria also leads to more development and growth of the GIT. Improvement of the intestinal morphology, such as increased villus height and villus height to crypt depth ratio, improves bird's performance by enhancing the digestion and absorption of nutrients^47^.

The body's first line of defense against pathogenic bacteria is the mucosal layer in the GIT^48^. Dysbacteriosis including *Salmonella* overgrowth may degrade this barrier, allowing bacteria to enter the circulation and causing systemic infections^49^. When *Salmonella* enters the circulation, it colonizes organs such as the liver, spleen, heart, kidneys and lungs^30,50^. We found that SE and coliform bacteria populations increased in the cecum and liver of challenged birds, suggesting mucosal barrier integrity was compromised. *Salmonella* colonization in the liver could cause hepatocyte necrosis, hepatitis, infiltration of heterophils and immune cells, congestion and hemorrhages in the liver of SE challenged birds, as previously reported^38,51,52^. Interestingly. the severity and score of these lesions were greater in the livers of the challenged compared to the exposed birds. Freitas Neto et al. (2007) also noted that *Salmonella* infections resulted in the most severe damage in the liver^53^.

Previous studies have suggested that liver lesions caused by bacterial incursions and serum biochemical profiles are correlated (REF). In our study, the increased activity of selected liver-derived enzymes such as ALT and AST in the serum confirmed the presence of liver lesions in the *Salmonella* challenged birds; which has been noted previously^54^. The loss of enzymes from the liver to the blood might be due to increased lipid peroxidation of hepatocytes due to inflammation caused by Lipopolysaccharides (LPS) of SE (endotoxin)^55,56^. Reduced serum albumin concentrations in the challenged chickens and control group may also be due to liver lesions as hypoalbuminemia is indicative of reduced liver function^38,57^. Increased serum globulin levels in the challenged chickens might be attributed to either stimulation of antigen production by the infectious agents or progression of liver disease and leakage of proteins^58^. In the challenged chickens, serum TG and cholesterol concentrations in the control group were significantly lower than those in the other treatments which is has also been noted in mycotoxicosis in chickens which resulted in similar hepatic dysfunction in broilers^59^. This could be explained by, either reduced feed intake or compromised lipid metabolism as a result of liver lesions^38^. Exposed chickens were not subject to the same problems as the lesions noted were likely not severe enough to cause significant dysfunction in lipid metabolism.

Villus growth is mainly induced by the maturation and migration of enterocytes, which, in turn, can increase the extent of digestion and absorption in the intestine^60^. Many reports have shown the anti-inflammatory role of *PPAR*γ, because *PPAR*γ ligands prevent large inflammation cascades like nuclear factor-κB (NF-κB)^61^. It has also been demonstrated that the dietary inclusion of *E*. *faecium* in chickens can reduce *TLR4* and *NF-κB* genes expression; however, it increased *PPARγ* gene expression^25,26^. It has also been shown that *PPARγ* could induce the differentiation of the mouse embryonic adipose tissue in vivo and in vitro^62^. Also, Ciglitazone, a *PPARγ* agonist, in the mouse neural stem cells (NSC) leads to neuro-glial differentiation through the induction of differentiation genes^63^. In addition, LPS is also one of the *PPARγ* agonists. We found that the exposed birds fed diets containing 0.3 g/kg colistin and 1.5 g/kg BP had numerically greater VH: CD and villus surface area (Table 5), and a lower expression of *PPARγ* which is associated with reduced inflammatory cascades at 14 DPC (Fig. 6B). The fact that changes in *PPARγ* expression was not correlated with VH: CD and villus surface area between treatments suggest that *PPARγ* is not involved in cell migration in the crypts^64^. BP supplementation likely reduced GIT inflammation by raising *PPAR* expression as has been shown to be the case in the regulation of bowel disease^65^.

Lipopolysaccharides, also known as endotoxins, are part of the outer membrane of Gram-negative bacteria; they are vital to maintaining the structural integrity of these microorganisms^66^. Lipopolysaccharides from Gram-negative bacteria like *E.* *coli* can trigger systemic and local immune responses^67^. In several studies, LPS has been used for triggering inflammation through the release of pro-inflammatory cytokines in the chicken^68–71^, by activation of *TLR4*^25^.

This study hypothesized that BP supplementation in SE-challenged broilers could affect the inflammation pathways by interacting with *PPARγ*, *TLR4* and *IL-10* gene expression. Based on the previous studies, we speculated that dietary supplementation with BP in the SE-challenged broilers might reduce *TLR4* expression due to reduced LPS concentrations. We also hypothesized that reducing *TLR4* expression would reduce *PPARγ* expression and reduce intestinal inflammation, cell differentiation**,** and cell migration. The major effects of BP on the GIT immune response were likely mediated through monocytes rather than cellular immunity.

During bacterial inflammation in the GIT, neutrophils are the first barrier against the inflammation inducer. LPS, for example, directly stimulate T regulatory cells, leading to *IL-10* transcription in the neutrophils; T regulatory cells, in turn, through the cell protein junctions (ICAM-1) with neutrophils (CD11b), induce *IL-10* secretion from neutrophils^72^ which reduces inflammation by inhibiting the proliferation of T cells^73^. Collectively, these studies have demonstrated that the disposal of several microorganisms, such as mycobacteria, Gram-negative and Gram-positive bacteria, can enforce *IL-10* production by neutrophils^74–78^. In addition, in vitro investigations have shown that *TLR4* agonist (LPS) potentially induces *IL-10* secretion in neutrophils^74^. The present study showed that supplementation with colistin or BP at the level of 1 g/kg numerically increased *IL-10* transcription in infected birds. Based on previous studies, increasing the activity of LPS-stimulated T regulatory cells could induce *IL-10* secretion from neutrophils. Also, *IL-10* production was increased through the positive feedback of *IL-10* on itself, regulating the immune response^72,79^.

In our study, the BP supplementation likely decreased Gram-negative bacteria, thus increasing the intestinal soluble peptidoglycans and lipoteichoic acid concentration and cell contact between T regulator cells and neutrophils; finally, it increased the IL-10 production by neutrophil, as demonstrated by Siepert et al. 2014^80^. We speculate that the low level (0.3 g/kg) of colistin supplementation could not decrease the number of harmful bacteria in the GIT, as reflected in the decreased IL-10 cytokine of the exposed chickens. LPS not only increased the secretions of pro-inflammatory cytokines from macrophages but also enhanced the secretion of anti-inflammatory cytokines from neutrophils^81,82^. However, in the infected chickens, BP supplementation at the lowest level not only increased the *IL-10* expression but also reduced the *TLR4* expression through the restrain of the pathogens.

The most common criticisms raised against phage therapy are as follows: (1) BP stimulates the production of neutralizing antibodies in the body; (2) during BP therapy, bacteria may become resistant to BP; and (3) BPs are only active when administered shortly after the bacterial infection^83^. The use of BPs may be associated with the risk of immunological reactions. Although bacteria are a typical host for BPs, BP can also interact with the immune cells^40^. Studies have shown that BP can get into the circulation regardless of how they are administered and if they do not find a bacterial host, they will be eliminated by phagocytic cells^84^. Moreover, the host immune system may produce antibodies (neutralizing antibodies) against BP^85,86^. which may be one of the most important factors in reducing the effectiveness of phage therapy^87^. The concentration of anti-phage neutralizing antibodies depends on the dose and route of BP administration; oral administration of BP slightly increases the antibody response. There are three approaches to solve these problems: repeating BP administration, increasing the BP dose and using different BPs^84,86^. In this study, we tried to prevent this problem by repeated administration of BP. Bacteria may become resistant to BP, which could hamper the effectiveness of BP therapy. However, bacterial resistance does not seem to be a problem for BP therapy as bacteria are about 10 times less resistant to BP than antibiotics^39,88^. In addition, the use of multiple BPs can reduce the development of bacterial resistance^40^. Naghizadeh et al. (2019) also reported that the use of a phage cocktail to control Colibacillosis was more effective than a single one due to the synergistic effects created among the individual phages^89^.

According to a previous study, BP therapy is effective when it is administered shortly after the infection^42^. Phage therapy has been shown to be more effective if the bird consumes BP before being exposed to the pathogen^90^; however, in reality, we do not know exactly when the bacteria contaminate the poultry flocks, and it takes several days for the infected birds to show clinical signs of disease. If BP therapy starts after observing signs of disease it will not have much effect, which is why it was included in the diet from the starter until the end of the grower period in the present Second, we found that BP could be effective on exposed chickens because they had relatively minor infections which were easier to control. Indeed, no systemic infection was detected in some of the exposed birds as SE was not isolated from their liver. Jeong et al. (2013) also reported that whereas BP effectively treated infected chickens, fewer exposed chickens were infected by transmitted *Salmonella* *gallinarum* when BP was added to the diet of the infected birds^30^. Our results showed that BP could be included in the diet as a dietary supplement to prevent bacterial infection and to reduce the spreading of the infection in the flock. Third, BP can act as a growth promoter supplement in the diet, even in the flocks, without any disease. In another experiment, we studied the effects of BP as a growth promoter supplement (under the normal condition and without bacterial challenge); we found that BP supplementation improved gut health and function by increasing the beneficial bacteria, improving the production performance of the broiler chickens^91^, which is in agreement with previous work in broilers and layers^92–96^.

We demonstrated that adding colistin to the diet of the exposed birds, all through their rearing period, was effective in controlling *Salmonella* infection. However, adding antibiotics to the diet is associated with risks. Antibiotics do not act specifically and may kill beneficial bacteria in the gut, leading to dysbacteriosis. On the other hand, it has been reported that adding antibiotics to the diet of birds reared under normal conditions and without any bacterial challenge may have detrimental effects on the intestinal cells; this is because, in such conditions, they have no bacterial host in the gut^97^. Therefore, they may destroy the intestinal villi and deteriorate the birds’ production performance. Lei et al. (2015) also added virginiamycin to the diet of the broiler chickens, reporting that virginiamycin shortened the villus height in the duodenum and jejunum^98^. In another experiment, we studied the effects of colistin as a growth promoter (AGP) in broiler chickens, finding that adding colistin to the diet of the birds from the first day of the rearing period reduced their growth performance^99^. Further, the use of antibiotics has been banned in many countries due to bacterial resistance. On the contrary, using BP is less risky; based on the results of the current study, they seem to be promising alternatives to antibiotics in controlling *Salmonella* in broiler chickens.

## Conclusions

Using BP to treat bacterial infections is not a new strategy; however, few studies have used BP as a supplement to prevent the spreading and transmission of *Salmonella* in birds. We demonstrated that BP could be used as a feed additive to increase growth performance and control *Salmonella* and other pathogenic bacteria in broilers. In addition, BP supplementation leads to the down-regulation of *TLR4* and *PPARγ* transcription genes and up-regulation of *IL-10* gene expression resulting in reduced GIT inflammation.

## Data Availability

The datasets used and/or analyzed during the current study are available from the corresponding author upon reasonable requests.

## References

[1] Lammerding AM (2006). Modeling and risk assessment for *Salmonella* in meat and poultry. J. AOAC Int..

[2] Salmon D, & Smith T, Report on swine plague. in *USDA**Bureau**of**Animal**Ind.**2nd**Annual**Report*. (USDA, 1885).

[3] Cosby DE (2015). Salmonella and antimicrobial resistance in broilers: A review. J. Appl. Poult. Res..

[4] Sofos, J.N., & Juneja, V.K. *Pathogens**and**Toxins**in**Food:**Challenges**and**Interventions*. (American Society for Microbiology, 2010).

[5] Kogut MH, Arsenault RJ (2015). A role for the non-canonical Wnt-β-catenin and TGF-β signaling pathways in the induction of tolerance during the establishment of a *Salmonella*
*enterica* serovar enteritidis persistent cecal infection in chickens. Front. Vet. Sci..

[6] Croft AC, D'Antoni AV, Terzulli SL (2007). Update on the antibacterial resistance crisis. Med. Sci. Monit..

[7] Apostolakos I, Piccirillo A (2018). A review on the current situation and challenges of colistin resistance in poultry production. Avian Pathol..

[8] Kempf I, Jouy E, Chauvin C (2016). Colistin use and colistin resistance in bacteria from animals. Int. J. Antimicrob. Agents..

[9] Kempf I (2013). What do we know about resistance to colistin in Enterobacteriaceae in avian and pig production in Europe?. Int. J. Antimicrob. Agents..

[10] Crhanova M (2011). Immune response of chicken gut to natural colonization by gut microflora and to Salmonella enterica serovar enteritidis infection. Infect. Immun..

[11] Gadde U (2017). Alternatives to antibiotics for maximizing growth performance and feed efficiency in poultry: A review. Anim. Health. Res. Rev..

[12] Kim JH (2018). Physiochemical treatment of feed and utilization of feed additives to control *Salmonella* in poultry. Food Sci. Anim. Resour..

[13] Twort FW (1915). An investigation on the nature of ultra-microscopic viruses. Lancet.

[14] Abedon ST (2014). Phage therapy: Eco-physiological pharmacology. Scientifica..

[15] Whichard JM, Sriranganathan N, Pierson FW (2003). Suppression of *Salmonella* growth by wild-type and large-plaque variants of bacteriophage Felix O1 in liquid culture and on chicken frankfurters. J. Food. Prot..

[16] Huff W (2003). Bacteriophage treatment of a severe *Escherichia*
*coli* respiratory infection in broiler chickens. Avian. Dis..

[17] Adhikari P (2017). Effect of dietary bacteriophage supplementation on internal organs, fecal excretion, and ileal immune response in laying hens challenged by *Salmonella* Enteritidis. Poult. Sci..

[18] Wernicki A, Nowaczek A, Urban-Chmiel R (2017). Bacteriophage therapy to combat bacterial infections in poultry. Virol. J..

[19] Toro H (2005). Use of bacteriophages in combination with competitive exclusion to reduce *Salmonella* from infected chickens. Avian. Dis..

[20] Oh JH, Park MK (2017). Recent trends in *Salmonella* outbreaks and emerging technology for biocontrol of *Salmonella* using phages in foods: A review. J. Microbiol. Biotechnol..

[21] Svircev A, Roach D, Castle A (2018). Framing the future with bacteriophages in agriculture. Viruses.

[22] Roach DR (2017). Synergy between the host immune system and bacteriophage is essential for successful phage therapy against an acute respiratory pathogen. Cell Host Microbe.

[23] Zepeda Cervantes J, Ramírez-Jarquín JO, Vaca L (2020). Interaction between virus-like particles (VLPs) and pattern recognition receptors (PRRs) from dendritic cells (DCs): Toward better engineering of VLPs. Front. Immunol..

[24] Carroll-Portillo A, Lin HC (2019). Bacteriophage and the innate immune system: Access and signaling. Microorganisms..

[25] Huang L (2019). Effects of the dietary probiotic, *Enterococcus*
*faecium* NCIMB11181, on the intestinal barrier and system immune status in *Escherichia*
*coli* O78-challenged broiler chickens. Probiot. Antimicrob. Proteins.

[26] Gadde UD (2017). Dietary *Bacillus*
*subtilis*-based direct-fed microbials alleviate LPS-induced intestinal immunological stress and improve intestinal barrier gene expression in commercial broiler chickens. Res. Vet. Sci..

[27] Hernandez-Patlan D (2019). Evaluation of the antimicrobial and intestinal integrity properties of boric acid in broiler chickens infected with *Salmonella*
*enteritidis*: Proof of concept. Res. Vet. Sci..

[28] Ashayerizadeh A (2017). Fermented rapeseed meal is effective in controlling *Salmonella*
*enterica* serovar *Typhimurium* infection and improving growth performance in broiler chicks. Vet. Microbiol..

[29] Biloni A (2013). Evaluation of effects of EarlyBird associated with FloraMax-B11 on *Salmonella* Enteritidis, intestinal morphology, and performance of broiler chickens. Poult. Sci..

[30] Jeong JP (2013). Therapeutic effects of bacteriophages against *Salmonella*
*gallinarum* infection in chickens. J. Microbiol. Biotechnol..

[31] Zhen W (2018). Effect of dietary *Bacillus*
*coagulans* supplementation on growth performance and immune responses of broiler chickens challenged by *Salmonella*
*enteritidis*. Poult. Sci..

[32] Gupta S (2005). Effect of ochratoxin A on broiler chicks challenged with *Salmonella*
*gallinarum*. Br. Poult. Sci..

[33] Mahdavi A (2010). Effects of dietary egg yolk antibody powder on growth performance, intestinal *Escherichia*
*coli* colonization, and immunocompetence of challenged broiler chicks. Poult. Sci..

[34] Ekim B (2020). Effects of *Paenibacillus*
*xylanexedens* on growth performance, intestinal histomorphology, intestinal microflora, and immune response in broiler chickens challenged with *Escherichia*
*coli* K88. Poult. Sci..

[35] Sakamoto K (2000). Quantitative study of changes in intestinal morphology and mucus gel on total parenteral nutrition in rats. J. Surg. Res..

[36] Prakatur I (2019). Intestinal morphology in broiler chickens supplemented with propolis and bee pollen. Animals.

[37] Babińska I (2012). Modulating effect of propolis and bee pollen on chicken breeding parameters and pathomorphology of liver and kidneys in the course of natural infection with *Salmonella*
*enteritidis*. Bull. Vet. Inst. Pulawy..

[38] Garcia KO (2010). Experimental infection of commercial layers using a Salmonella enterica sorovar Gallinarum strain: Blood serum components and histopathological changes. Braz. J. Vet. Pathol..

[39] Ly-Chatain MH (2014). The factors affecting effectiveness of treatment in phages therapy. Front. Microbiol..

[40] Nilsson AS (2014). Phage therapy—Constraints and possibilities. Ups. J. Med. Sci..

[41] Azizian R, Nasab SDM, Ahmadi NA (2013). Bacteriophage as a novel antibacterial agent in industry and medicine. J. Paramed. Sci..

[42] Bardina C (2014). Significance of the bacteriophage treatment schedule in reducing *Salmonella* colonization of poultry. Appl. Environ. Microbiol..

[43] Parveen S (2017). Reduction of *Salmonella* in ground chicken using a bacteriophage. Poult. Sci..

[44] Fiorentin L, Vieira ND, Barioni W (2005). Oral treatment with bacteriophages reduces the concentration of *Salmonella* Enteritidis PT4 in caecal contents of broilers. Avian. Pathol..

[45] Kogut MH (2019). The effect of microbiome modulation on the intestinal health of poultry. Anim. Feed. Sci. Technol..

[46] Pan D, Yu Z (2014). Intestinal microbiome of poultry and its interaction with host and diet. Gut Microbes..

[47] Yadav S, Jha R (2019). Strategies to modulate the intestinal microbiota and their effects on nutrient utilization, performance, and health of poultry. J. Anim. Sci. Biotechnol..

[48] Brisbin JT, Gong J, Sharif S (2008). Interactions between commensal bacteria and the gut-associated immune system of the chicken. Anim. Health. Res. Rev..

[49] Wang H (2014). Intestinal dysbacteriosis contributes to decreased intestinal mucosal barrier function and increased bacterial translocation. Lett. Appl. Microbiol..

[50] Suzuki S (1994). Pathogenicity of *Salmonella*
*enteritidis* in poultry. Int. J. Food. Microbiol..

[51] Shivaprasad H (2000). Fowl typhoid and pullorum disease. Rev. Sci. Tech..

[52] Christensen J (1996). Correlation between viable counts of *Salmonella* Gallinarum in spleen and liver and the development of anaemia in chickens as seen in experimental fowl typhoid. Avian. Pathol..

[53] Freitas Neto O (2007). Infection of commercial laying hens with *Salmonella* Gallinarum: Clinical, anatomopathological and haematological studies. Rev. Bras. Cienc. Avic..

[54] Rocha TM (2013). Liver function and bacteriology of organs in broiler inoculated with nalidixic acid-resistant *Salmonella*
*typhimurium* and treated with organic acids. Ital. J. Anim. Sci..

[55] Benzer F (2009). Influence of enrofloxacin administration on oxidative stress and antioxidant enzyme activities of experimentally infected broilers with *Salmonella*
*enterica* serovar enteritidis. Vet. Sci..

[56] Hamid F, El-Gohary E, Risha E (2013). Incorporation efficacy comparison of probiotic and antibiotic on growth performance, some immunological and biochemical parameters in *Ealmonella*
*entertidis* challenged chicks. Life Sci. J..

[57] Fotouh A (2014). Alterations of blood components in broiler chicks experimentally infected with *Salmonella* Gallinarum. Glob. Vet..

[58] Abd-El-Rahman AH (2012). Effect of Bactocell and revitilyte-plus as probiotic food supplements on tm the growth performance, hematological, biochemical parameters and humoral immune response of Broiler Chickens. Sci J..

[59] Aravind K (2003). Efficacy of esterified glucomannan to counteract mycotoxicosis in naturally contaminated feed on performance and serum biochemical and hematological parameters in broilers. Poult. Sci..

[60] Kim J (2012). Effect of supplementation of multi-microbe probiotic product on growth performance, apparent digestibility, cecal microbiota and small intestinal morphology of broilers. J. Anim. Physiol. Anim. Nutr..

[61] Zhang L (2017). Ginsenoside Rg1 attenuates adjuvant-induced arthritis in rats via modulation of PPAR-γ/NF-κB signal pathway. Oncotarget.

[62] Rosen ED (1999). PPARγ is required for the differentiation of adipose tissue in vivo and in vitro. Mol. Cell..

[63] Kanakasabai S (2012). PPARγ agonists promote oligodendrocyte differentiation of neural stem cells by modulating stemness and differentiation genes. PLoS ONE.

[64] Necela BM, Thompson EA (2008). Pathophysiological roles of PPAR γ in gastrointestinal epithelial cells. PPAR Res..

[65] Adachi M (2006). Peroxisome proliferator activated receptor γ in colonic epithelial cells protects against experimental inflammatory bowel disease. Gut.

[66] Sutcliffe IC (2010). A phylum level perspective on bacterial cell envelope architecture. Trends Microbiol..

[67] Wang L (2015). Methods to determine intestinal permeability and bacterial translocation during liver disease. J. Immunol. Methods.

[68] Tan J (2014). Dietary l-arginine supplementation attenuates lipopolysaccharide-induced inflammatory response in broiler chickens. Br. J. Nutr..

[69] Zhang M (2012). Expression of Toll-like receptors and effects of lipopolysaccharide on the expression of proinflammatory cytokines and chemokine in the testis and epididymis of roosters. Poult. Sci..

[70] Munyaka P (2012). Immunomodulation in young laying hens by dietary folic acid and acute immune responses after challenge with *Escherichia*
*coli* lipopolysaccharide. Poult. Sci..

[71] Keestra AM, van Putten JP (2008). Unique properties of the chicken TLR4/MD-2 complex: Selective lipopolysaccharide activation of the MyD88-dependent pathway. J. Immunol..

[72] Lewkowicz N (2016). Induction of human IL-10-producing neutrophils by LPS-stimulated Treg cells and IL-10. Mucosal Immunol..

[73] Dima AA (2011). Comparison of segmentation algorithms for fluorescence microscopy images of cells. Cytometry A.

[74] Zhang X (2009). Coactivation of Syk kinase and MyD88 adaptor protein pathways by bacteria promotes regulatory properties of neutrophils. Immunity.

[75] Tsuda Y (2004). Three different neutrophil subsets exhibited in mice with different susceptibilities to infection by methicillin-resistant *Staphylococcus*
*aureus*. Immunity.

[76] Bouabe H (2011). Novel highly sensitive IL-10–β-lactamase reporter mouse reveals cells of the innate immune system as a substantial source of IL-10 in vivo. J. Immunol..

[77] Ocuin LM (2011). Neutrophil IL-10 suppresses peritoneal inflammatory monocytes during polymicrobial sepsis. J. Leukoc. Biol..

[78] Greenblatt MB (2010). Calcineurin regulates innate antifungal immunity in neutrophils. J. Exp. Med..

[79] Sanjabi S (2009). Anti-inflammatory and pro-inflammatory roles of TGF-β, IL-10, and IL-22 in immunity and autoimmunity. Curr. Opin. Pharmacol..

[80] Siepert B (2014). Enterococcus faecium NCIMB 10415 supplementation affects intestinal immune-associated gene expression in post-weaning piglets. Vet. Immunol. Immunopathol..

[81] Waseem T (2008). Exogenous ghrelin modulates release of pro-inflammatory and anti-inflammatory cytokines in LPS-stimulated macrophages through distinct signaling pathways. Surgery..

[82] Liu CJ, Lin JY (2012). Anti-inflammatory and anti-apoptotic effects of strawberry and mulberry fruit polysaccharides on lipopolysaccharide-stimulated macrophages through modulating pro-/anti-inflammatory cytokines secretion and Bcl-2/Bak protein ratio. Food Chem. Toxicol..

[83] Capparelli R (2010). Bacteriophage therapy of Salmonella enterica: A fresh appraisal of bacteriophage therapy. J. Infect. Dis..

[84] Cisek AA (2017). Phage therapy in bacterial infections treatment: one hundred years after the discovery of bacteriophages. Curr. Microbiol..

[85] Dabrowska K (2005). Bacteriophage penetration in vertebrates. J. Appl. Microbiol..

[86] Górski, A. *et**al*. Phage as a modulator of immune responses: Practical implications for phage therapy. in *Advances**in**Virus**Research*. 41–71 (Elsevier, 2012). 10.1016/B978-0-12-394438-2.00002-5 22748808

[87] Smith HW, Huggins MB, Shaw KM (1987). Factors influencing the survival and multiplication of bacteriophages in calves and in their environment. Microbiology.

[88] Carlton RM (1999). Phage therapy: Past history and future prospects. Arch. Immunol. Ther. Exp. (English edition).

[89] Naghizadeh M (2019). Synergistic effect of phage therapy using a cocktail rather than a single phage in the control of severe colibacillosis in quails. Poult. Sci..

[90] Clavijo V, Flórez MJV (2018). The gastrointestinal microbiome and its association with the control of pathogens in broiler chicken production: A review. Poult. Sci..

[91] Sarrami Z, Sedghi M, Mohammadi I, Kim WK, Mahdavi AH (2022). Effects of bacteriophage supplement on the growth performance, microbial population, and PGC-1α and TLR4 gene expressions of broiler chickens. Sci. Rep..

[92] Kim J (2015). Effect of dietary supplementation of bacteriophage on performance, egg quality and caecal bacterial populations in laying hens. Br. Poult. Sci..

[93] Kim J (2014). Effect of dietary supplementation of bacteriophage on growth performance and cecal bacterial populations in broiler chickens raised in different housing systems. Livest. Sci..

[94] Wang J (2013). Evaluation of bacteriophage supplementation on growth performance, blood characteristics, relative organ weight, breast muscle characteristics and excreta microbial shedding in broilers. Asian-Australas J. Anim. Sci..

[95] Zhao P, Baek H, Kim I (2012). Effects of bacteriophage supplementation on egg performance, egg quality, excreta microflora, and moisture content in laying hens. Asian-Australas J. Anim. Sci..

[96] Noor M, Runa N, Husna A (2020). Evaluation of the effect of dietary supplementation of bacteriophage on production performance and excreta microflora of commercial broiler and layer chickens in Bangladesh. MOJ Proteom. Bioinform..

[97] Chichlowski M (2007). Microarchitecture and spatial relationship between bacteria and ileal, cecal, and colonic epithelium in chicks fed a direct-fed microbial, PrimaLac, and salinomycin. Poult. Sci..

[98] Lei X (2015). Effect of Bacillus amyloliquefaciens-based direct-fed microbial on performance, nutrient utilization, intestinal morphology and cecal microflora in broiler chickens. Asian-Australas J. Anim. Sci..

[99] Sedghi M, Mohammadi I, Sarrami Z (2022). Effects of a yeast cell wall product on the performance of broiler chickens and PGC-1α, TLR4, IL-10 and PPARγ genes expression. Ital. J. Anim. Sci..

[100] Khan S, Roberts J, Wu SB (2017). Reference gene selection for gene expression study in shell gland and spleen of laying hens challenged with infectious bronchitis virus. Sci. Rep..

[101] Wang G, McConn BR, Liu D (2017). The effects of dietary macronutrient composition on lipid metabolism-associated factor gene expression in the adipose tissue of chickens are influenced by fasting and refeeding. BMC Obes..

[102] Asgari F, Falak R, Teimourian S (2018). Effects of oral probiotic feeding on toll-like receptor gene expression of the chicken’s cecal tonsil. Rep. Biochem. Mol. Biol..

[103] Lee, R., Jung, J.S., Yeo, J. *et**al*. Analysis of immune response of chicken primary cells by infection with Korean IBV strain. *Res**Sq*. **3**, 1–11 (2021).

